# Development and evaluation of an up-converting phosphor technology-based lateral flow assay for rapid detection of *Francisella tularensis*

**DOI:** 10.1038/srep17178

**Published:** 2015-11-26

**Authors:** Fei Hua, Pingping Zhang, Fuli Zhang, Yong Zhao, Chunfeng Li, Chongyun Sun, Xiaochen Wang, Ruifu Yang, Chengbin Wang, Ailian Yu, Lei Zhou

**Affiliations:** 1 Laboratory of Analytical Microbiology, State Key Laboratory of Pathogen and Biosecurity, Beijing Institute of Microbiology and Epidemiology, Beijing 100071, P.R. China; 2 Beijing Key Laboratory of POCT for Bioemergency and Clinic (No. BZ0329), Beijing 100071, P.R. China; 3 Department of Etiology, Taishan Medical University, Taian 271000, P.R. China; 4 Clinical Diagnostic Center, 302nd Hospital of the People’s Liberation Army, Beijing 100039, P.R. China; 5 Radiation Oncology Department, Beijing Military General Hospital, Beijing 100700, P.R. China; 6 Department of Clinical Laboratory, Chinese People’s Liberation Army General Hospital, Beijing 100853, P.R. China; 7 College of Animal Science and Technology, Jilin Agricultural University, Changchun 130118, P.R. China

## Abstract

*Francisella tularensis* is a potential biowarfare/bioterrorism agent and zoonotic pathogen that causes tularemia; thus, surveillance of *F. tularensis* and first-level emergency response using point-of-care testing (POCT) are essential. The UPT-LF POCT assay was established to quantitatively detect *F. tularensis* within 15 min, and the sensitivity of the assay was 10^4^ CFU · mL^−1^ (100 CFU/test). The linear quantitative range covered five orders of magnitude, and the coefficients of variation were less than 10%. Except *Shigella dysenteriae*, UPT-LF showed excellent specificity to four strains that are also potential biowarfare/bioterrorism agents and 13 food-borne pathogenic strains. Samples with pH 2–13, high ion strengths (≥2 mol · L^−1^ solution of KCl and NaCl), high viscosities (≤50 mg · mL^−1^ PEG20000 or ≥20% glycerol), and high concentrations of biomacromolecules (≥400 mg · mL^−1^ bovine serum albumin or ≥80 mg · mL^−1^ casein) showed little influence on the assay. For practical utilization, the tolerance limits for seven powders and eight viscera were determined, and operation errors of liquid measurement demonstrated a minor influence on the strip. Ftu-UPT-LF is a candidate POCT method because of its excellent sensitivity, specificity, and stability in complex samples, as well as low operation error.

Tularemia is a serious infectious zoonotic disease of the northern hemisphere, such as in North America, Europe, and North Asia. Although antibiotic treatment is effective in infectious patients, the high misdiagnosis rate causes a great deal of confusion and becomes an obstacle to disease prevention. The six main clinical symptoms, including oropharyngeal form, ulceroglandular form, pneumonic form, and typhoidal form, are often misdiagnosed as influenza because they all begin with fever^1^. Moreover, these clinical symptoms depend on various routes of infection, making diagnosis difficult. For example, the pneumonic form caused by *Francisella tularensis* exhibits no clinical characteristics to distinguish it from pneumonia of other causes^2^.

*F. tularensis*, the pathogen causing tularemia, has four recognized subspecies, namely, *F. tularensis* subsp. *Tularensis* (type A), *holarctica* (type B), *mediasiatica*, and *novicida*. Among these subspecies, type A and type B cause the majority of reported cases of tularemia in humans, with type A causing more severe cases. Type A is regarded as a category A biowarfare/bioterrorism agent^3^ because of the diversity of its route of transmission, ease of dissemination (especially the aerosol route), high infectivity, and potentially high mortality rate^4^. To date, *F. tularensis* can be isolated from 250 kinds of animals, such as fish, bird, mammal (rodent and lagomorpha), and arthropods^5^. In addition, *F. tularensis* can survive for several months in extreme conditions, such as salty environments or environments with low temperatures and saline (e.g., frozen water, soil, and milk). Humans are usually infected by inhalation of aerosolized bacteria, handling of infected animals, arthropod bites, and ingestion of contaminated food or water^6^,^7^,^8^. *F. tularensis* in the environment can be eliminated simply by heating the bacteria at 60 °C for 20 min or using ordinary medical disinfectants. Thus, surveillance in animals and first-level emergency response in biowarfare and bioterrorism may prevent or minimize outbreaks in humans, and point-of-care testing (POCT) plays a key role in early accurate diagnosis and screening.

For optimal use in low-resource setting, POCT must be rapid, sensitive, simple, easily interpretable, and stable under extreme conditions^9^. The detection of *F. tularensis* from complex practical samples, such as suspicious animal samples and “white” powders, by a nonprofessional is inevitable. Antibodies often develop late in the course of tularemia. After they are produced, high titers of both IgG and IgM can persist for more than 10 years^2^,^10^, thereby causing serological tests (agglutination assays) to generate false negative results in the initial stages of tularemia or reflect a previous infection, similar to enzyme-linked immunosorbent assay (ELISA). For pathogen detection in real samples (e.g., food, viscera, and soil), some complicated components in samples may easily influence the detection results for ELISA and nucleic acid tests (such as sequencing and polymerase chain reaction); therefore, sample pretreatment and complicated operations are often required for complex samples^11^,^12^.

Lateral flow assay is a candidate POCT in low-resource setting because it is rapid, relatively inexpensive, easily interpretable, and stable^13^,^14^. However, traditional bio-labels, such as colloidal gold particles, display results observed by naked eyes, leading to low sensitivity. Up-converting phosphor technology-based lateral flow (UPT-LF) assay uses new bio-labels, the up-converting phosphor particles (UCPs) with unique luminescent property^15^. Its optical signals can be collected and transformed into electrical signals, so the target is detected quantitatively with high sensitivity. UPT-LF assay has also been successively utilized for the detection of parasites^16^ and bacteria^17^,^18^,^19^. Given UCP’s stable optical properties and strong covalent combination with antibodies, UPT-LF assay shows robust performance for many practical samples, such as blood, urine, and saliva^16^,^20^. This assay is especially suitable for the surveillance of natural foci and first-level emergency responses in biowarfare and bioterrorism, because it is tolerant to highly complex samples (such as viscera collected from surveillance and various “white powders” present in anti-terrorist applications) and presents low operation error caused by nonprofessionals^17^,^18^. Thus, UPT-LF may be a feasible method for on-site detection of *F. tularensis*.

In this study, specific antibodies were covalently bound to the UCP reporter particle to establish the UPT-LF assay for *F. tularensis*. The assay’s performance was comprehensively evaluated in actual application, particularly its tolerance for multiplex samples and the operation error.

## Results and Discussion

### Establishment of UPT-LF assay for the detection of *F. tularensis*

The UPT-LF strip was fabricated as previously described^18^,^19^. Monoclonal antibodies (mAbs) on the strip were prepared through subcutaneous injection with inactivated *F. tularensis* and then screened by ELISA coated with the bacteria. The conjugate release pads of UCP combined with different mAbs, as well as nitrocellulose membranes coated with various mAbs, were matched and assembled into the strips. In this study, the signal peak areas at the test line and control line on the strip were defined as T and C values, whereas the T/C ratio was employed as the result of the detection^15^,^20^. The optimal matches with significant differences in T/C ratios between the negative and positive samples were designated in the prepared strips for *F. tularensis* detection. Such strips were named Ftu-UPT-LF. The sample-treating buffer for Ftu-UPT-LF was further optimized to intensify the differences in T/C ratios between negative and positive samples, and finally it was determined as 0.03 mol · L^−1^ phosphate buffer (PB) containing 0.5% NP-40 and 0.25 mol · L^−1^ NaCl.

### Sensitivity, linearity, and precision

**Figure 1:**
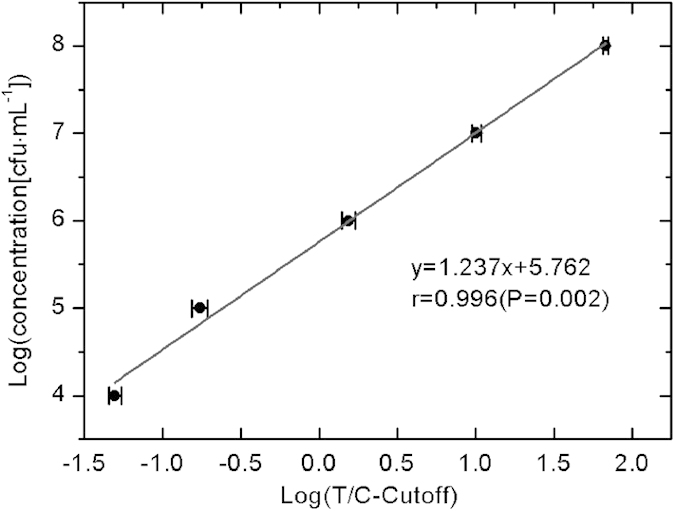
Standard quantification curve of Ftu-UPT-LF obtained by *Francisella tularensis* diluted by phosphate buffer. The sensitivity of Ftu-UPT-LF was 10^4^ CFU · mL^−1^. The coefficients of variation of the three tests were less than 10%.

PB was detected 10 times as the blank control, and the mean ± 3 SD of T/C ratios was set as the cutoff threshold. The T/C ratios of *F. tularensis* diluted by PB ranging from 10^3^ CFU · mL^−1^ to 10^9^ CFU · mL^−1^ were measured and set as the criterion to evaluate the influence of different interference factors. The lowest concentration with higher T/C ratios than the cutoff threshold was defined as sensitivity, and the sensitivity of Ftu-UPT-LF was 10^4^ CFU · mL^−1^. The standard quantification curve was plotted with the logarithm of the T/C-cutoff value as *x* and the logarithm of concentration as *y* (Fig. 1). The correlation coefficient of linear regression analysis was 0.996, which indicated that Ftu-UPT-LF demonstrated high accuracy for *F. tularensis* with a concentration range of 10^4^–10^8^ CFU · mL^−1^. The coefficients of variation for all samples were all less than 10%, demonstrating excellent precision of quantification.

### Specificity

**Figure 2:**
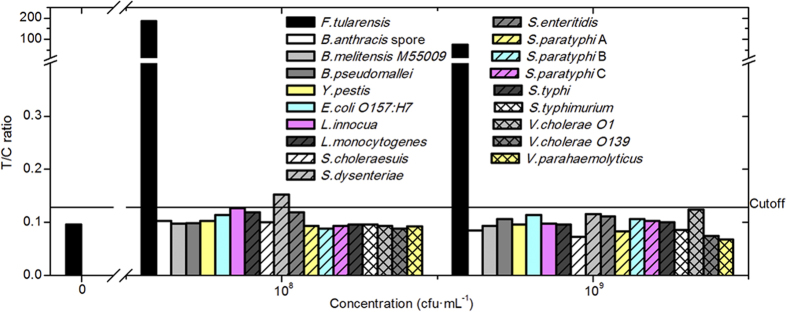
Specificity assessments of Ftu-UPT-LF. Except *Shigella dysenteriae*, Ftu-UPT-LF showed excellent specificity for 10^8^ and 10^9^ CFU · mL^−1^ of four strains as potential biowarfare/bioterrorism agents and 13 food-borne pathogenic bacterial strains with similar gastrointestinal infectious routes to *F. tularensis*.

Four strains as potential biowarfare/bioterrorism agents and 14 food-borne pathogenic bacterial strains with similar gastrointestinal infectious routes to *F. tularensis* were chosen to evaluate the specificity of Ftu-UPT-LF. Except *Shigella dysenteriae*, non-reactive samples were observed for these bacterial strains even at 10^8^ and 10^9^ CFU · mL^−1^ (Fig. 2).

Additionally, some strains, including *F. tularensis*, showed high T/C ratios at 10^8^ CFU · mL^−1^ compared with those at 10^9^ CFU · mL^−1^. For example, 10^9^ CFU · mL^−1^ *S. dysenteriae* did not influence Ftu-UPT-LF but destroyed the specificity at 10^8^ CFU · mL^−1^. High bacterial concentrations possibly clogged the pores on the strips and blocked the reactions among antibodies and antigens. The signals at the T line declined more obviously than those at the C line because the T line was closer to the sample, thereby leading to a decrease in the T/C ratios at high bacterial concentrations. This phenomenon was especially obvious for *F. tularensis*, in which the T/C ratios decreased more sharply at 10^9^ CFU · mL^−1^ than that at 10^8^ CFU · mL^−1^ (Fig. 2).

### Sample tolerance of Ftu-UPT-LF

As a zoonotic pathogen and biowarfare/bioterrorism agent, *F. tularensis* might be present in the animals’ viscera, soil, or various “white powders.” Variations in pH, ion strength, viscosity, and biological matrices of these samples might influence the reaction between antibodies and antigens on the surface of bacteria or slow the flow of liquid through the strip, leading to false positive and false negative results. However, the components of real samples were too complicated to analyze their influence on Ftu-UPT-LF. To simplify this problem, prior to the evaluation of real sample tolerance, the chemical and biological agents with those properties were spiked by *F. tularensis* and then applied to the strip to explore the single-factor theoretical tolerance of Ftu-UPT-LF.

Ftu-UPT-LF was defined as tolerant to a specific influencing factor on the condition that the specificity and sensitivity of the strip were maintained under its influence (i.e., the T/C ratio of the negative sample was lower than the cutoff threshold, and that of the positive sample was higher than the cutoff). The highest concentration of a specific influencing factor that the strip could tolerate was defined as the tolerance limit, and the tolerance limits of all the samples are listed in Table 1.

### Evaluation of single-factor theoretical tolerance

**Figure 3:**
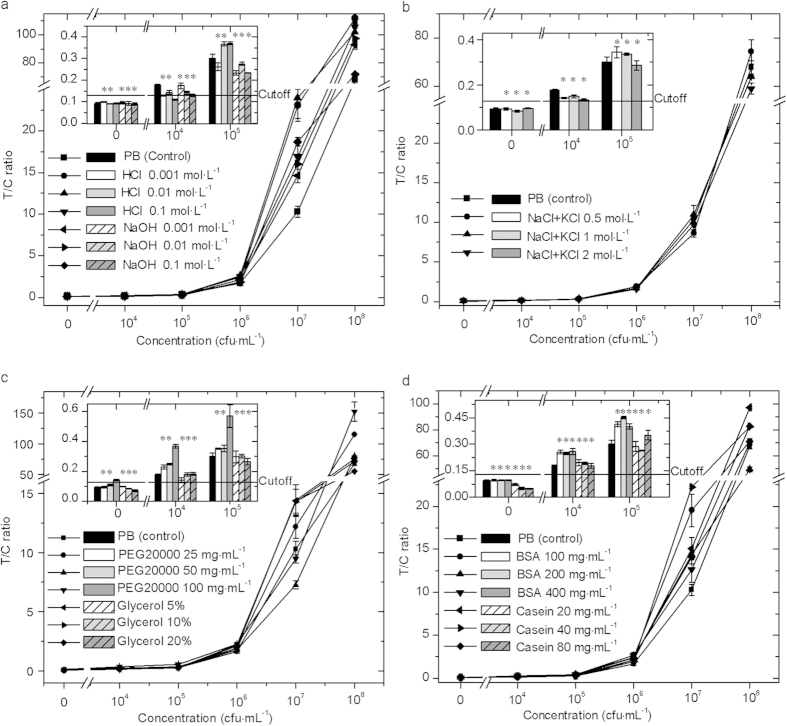
T/C ratios of Ftu-UPT-LF under the influence of (**a**) pH, (**b**) ion strength, (**c**) viscosity, and (**d**) biological matrices. The line graphs represent the T/C ratios of all samples with *F. tularensis* ranging from 10^4^ CFU · mL^−1^ to 10^8^ CFU · mL^−1^, whereas the bar graphs show the results of negative and weak positive samples in detail. Ftu-UPT-LF maintained sensitivity and specificity (*) at certain concentrations of the single-factor interference.

Serial concentrations of HCl and NaOH, the mixture of KCl and NaCl, PEG20000 and glycerol, and BSA and casein were used to assess the single-factor theoretical tolerance of Ftu-UPT-LF to pH, ion strength, viscosity, and biological matrices, respectively. Under the influence of various chemical and biological agents, the T/C ratios of all samples with *F. tularensis* ranging from 10^4^ CFU · mL^−1^ to 10^8^ CFU · mL^−1^ showed favorable consistency with that of the control (line graph in Fig. 3). The error in quantitative analysis of Ftu-UPT-LF was less than one order of magnitude, indicating the accuracy of quantitative detection.

The T/C ratios of the negative and weak positive samples under the influence of various chemical and biological agents are shown in detail as a bar graph in Fig. 3. HCl and NaOH decreased the T/C ratios, whereas 0.1 mol · mL^−1^ HCl destroyed the sensitivity of the strip, so Ftu-UPT-LF could tolerate the solution with pH 2–13. HCl with high concentrations may affect the sensitivity of Ftu-UPT-LF in many different ways. On the one hand, parts of targeted antigens on the surface of bacterial cells may be destroyed with cell lysis. On the other hand, the antigen–antibody reaction on the strip was affected by the mixture applied on the LF strip, which could not be neutralized by the sample-treating buffer. Saline solution slightly decreased the T/C ratios, and the tolerance limit was above 2 mol · mL^−1^. As viscous matter, PEG20000 and glycerol showed different influences on Ftu-UPT-LF, whereas similar phenomena were found in BSA and casein, which are both biological matrices. As macromolecules, PEG20000 and BSA remarkably increased the T/C ratios compared with the control, and their tolerance limit could reach 50 and above 400 mg · mL^−1^ respectively. As micromolecules, glycerol and casein significantly decreased the T/C ratios of negative samples, and their tolerance limit could reach above 20% and above 80 mg · mL^−1^. Apart from the expected influences, macromolecular matters possibly have other unique effects on antibodies fabricated on the strips, leading to different influences on Ftu-UPT-LF compared with micromolecules. Aside from being a viscous material, PEG can also react with the hydrophilic group of antibodies and impede the reaction between antibodies and water in space, so more hydrophobic antibodies have the opportunity to react with antigens on the surface of bacteria and increase the signals. As a macromolecule protein, BSA can effectively increase the concentration of proteins, preventing the degradation of antibodies on the strip.

Additionally, changes in the T/C ratios for *F. tularensis* at low and high concentrations differed under certain influencing factors. For example, under the influence of HCl and NaOH, the T/C ratios of the weak positive samples decreased, but that of positive samples with *F. tularensis* above 10^6^ CFU · mL^−1^ remarkably increased. The same result was observed for casein. These findings suggested that the agents exerted complicated effects on Ftu-UPT-LF for the accuracy of quantitation of *F. tularensis*. Single-factor agents may influence Ftu-UPT-LF in two important aspects. (i) The target bacteria may be lysed by some agents. More target antigens on the surface of bacteria were released, resulting in the increase in T/C ratios, or the target antigens of bacteria were also destroyed, resulting in the decrease in T/C ratios. (ii) The reaction between antibodies on the strip and antigens on the surface of bacteria may be enhanced or inhibited by the agents, resulting in the increase or decrease in T/C ratios. These effects could be added together to influence the quantitative results for *F. tularensis*.

### Evaluation of the influence of real samples

**Figure 4:**
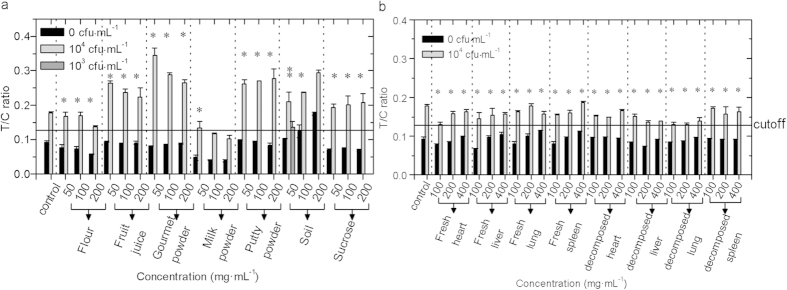
Tolerance of Ftu-UPT-LF to the real sample. Ftu-UPT-LF maintained sensitivity and specificity (*) and even improved the sensitivity by 10-fold (**) under the influence of powders and viscera of gradient concentrations.

The “white” powders in terrorist attacks (including flour, fruit juice, gourmet powder, milk powder, putty powder, and sucrose), environmental material (soil), and viscera present in the surveillance center (including fresh and decomposed states) were prepared into various concentrations of solutions or homogenates to evaluate the tolerance of Ftu-UPT-LF. As Fig. 4 shows, Ftu-UPT-LF could tolerate 50, 100, and 200 mg · mL^−1^ flour, fruit juice, gourmet powder, putty powder, and sucrose. With increasing concentration, milk powder decreased the T/C ratios and destroyed the sensitivity of Ftu-UPT-LF at 100 mg · mL^−1^. By contrast, soil increased the T/C ratios, resulting in a 10-fold improvement in sensitivity at 50 mg · mL^−1^, and the specificity was destroyed at 200 mg · mL^−1^. Ftu-UPT-LF showed excellent tolerance to all viscera with concentrations ranging from 100 mg · mL^−1^ to 400 mg · mL^−1^.

### Evaluation of the influence of operation error

**Figure 5:**
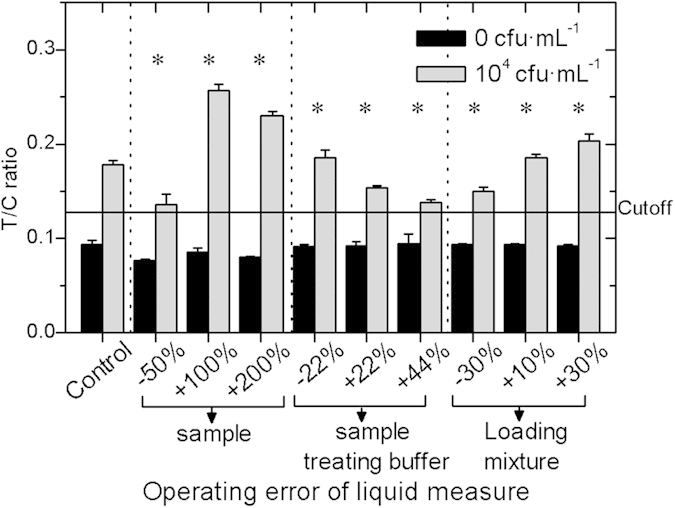
Tolerance of Ftu-UPT-LF to operation error. Ftu-UPT-LF maintained the sensitivity and specificity under the operation error of sample (from −50% to +200%), sample-treating buffer (from −22% to +44%), and loading mixture measure (from −30% to +30%).

As a POCT assay, Ftu-UPT-LF must be tolerant to the operation error caused by nonprofessionals during field application. The operation errors for UPT-LF were mainly from the liquid measurements, including the measurements of samples, sample-treating buffer, and loading mixture. Thus, we adjusted their volumes to evaluate the influence of operation errors on Ftu-UPT-LF. As shown in Fig. 5, when the deviation rates of the sample, sample-treating buffer, and loading mixture were −5% to +200%, −22% to +44%, and −30% to +30%, respectively, Ftu-UPT-LF could still maintain its sensitivity and specificity. Given that the T/C ratios depend on the actual bacterial content, the increase in the sample volume ratio raised the T/C ratios, whereas the increase in sample-treating buffer led to sharp falls in the T/C ratios.

On the basis of our previous analysis, when the ratio of sample and sample-treating buffer was invariable, the increased loading mixture had no significant influence on the T/C ratios of the strips, because only a slight variation in the effective flux increment of loading mixture flowing through the detection line was found because of the limited bed volume of absorbent pad and gradually slowing flow rate on nitrocellulose filter^18^. On the contrary, the increased loading mixture raised the T/C ratios of Ftu-UPT-LF for the positive samples because the signals at the T and C lines both decreased, and the signals at the C line decreased more obviously. The possible reason for this phenomenon is that the increasing applied loading mixture might influence the flow rate of the strip, and the bonds between antibodies against *F. tularensis* and antigens (or goat anti-mouse antibodies) might be more susceptible to variations in flow rate compared with the bonds in other kinds of UPT-LF strips.

## Conclusion

We established the UPT-LF POCT assay for the detection of *F. tularensis*, and its sensitivity reached 10^4^ CFU · mL^−1^ (100 CFU/test). The reagents with a wide pH range (2–13), high ion strengths, high viscosity, and high concentration of biomacromolecules, which might have a significant influence on the immunological assay, were applied to Ftu-UPT-LF. The sensitivity of the assay was maintained, and the deviation for quantitation was less than one order of magnitude. For practical utilization, *F. tularensis* could be directly detected using Ftu-UPT-LF by a nonprofessional within 15 min from complex samples merely through dissolving or grinding the samples, in contrast to other methods that require complicated sample pretreatment. The advantages of Ftu-UPT-LF are mainly derived from UCPs, which have stable optical property, especially the up-converting luminescence mechanism that eliminates the interference of background fluorescence. The covalent bond between UCP and antibodies was strong and could not be easily influenced by the complicated components in real samples applied on the strip. In summary, the UPT-LF POCT assay for *F. tularensis* detection is sensitive, rapid, inexpensive, easily interpretable, stable, and tolerant to complex samples, with low operation error. It is applicable for surveillance of natural foci and first-level emergence response in biowarfare and bioterrorism.

## Methods

### Ethics statement

All experiments were performed in accordance with the Guidelines for the Welfare and Ethics of Laboratory Animals of China. All experimental protocols were approved by the Committee of the Welfare and Ethics of Laboratory Animals, Beijing Institute of Microbiology and Epidemiology (Beijing, China). Eight-week-old female Balb/c mice were obtained from the Laboratory Animal Research Center, Academy of Military Medical Sciences (China). Mice acquisition was granted license by the Ministry of Health in the General Logistics Department of Chinese People’s Liberation Army.

### Reagents

UCP (NaYF4:Yb^3+^,Er^3+^) with excitation and emission spectrum peaks of 980 and 541.5 nm, respectively, was prepared by Dr. Yan Zheng from Shanghai Kerune Phosphor Technology Co., Ltd. (Shanghai, China). Nitrocellulose membrane (SHE 1350225) and glass fiber (GFCP20300) were purchased from Millipore Corp. (Bedford, MA, USA). Absorbent papers (Nos 470 and 903) were obtained from Schleicher & Schuell, Inc. (Keene, NH, USA). Plastic cartridges were designed by our group and processed by Shenzhen Jincanhua Industry Co. (Shenzhen, China).

Formaldehyde, HCl, NaOH, KCl, NaCl, PEG20000, glycerin, bovine serum albumin V (BSA), and casein were of analytical grade and purchased from Sigma–Aldrich (St. Louis, MO, USA). The real samples, including flour, fruit juice, gourmet powder, milk powder, putty powder, and sucrose, were all obtained from the local market, and soil samples were excavated from a parterre. Viscera obtained from Balb/C mice, including heart, liver, lung, and spleen, were divided into two parts. One part was stored at −20 °C as fresh specimen, and the other part was incubated at 37 °C for two weeks as decomposed specimen.

### Bacterial culture and mAb preparation

*F. tularensis* subsp. *holarctica* live vaccine strain was used for specific detection. The bacterial strains used for specificity evaluation were *Bacillus anthracis* Spore, *Brucella melitensis* M55009, *Burkholderia pseudomallei*, *Escherichia coli* O157:H7, *L. innocua*, *L. monocytogenes*, *Salmonella choleraesuis*, *S. dysenteriae*, *S. enteritidis*, *Salmonella paratyphi* A, *S. paratyphi* B, *S. paratyphi* C, *Salmonella typhi*, *Salmonella typhimurium*, *Vibrio cholerae* O1, *V. cholera* O139, *Vibrio parahaemolyticus*, and *Yersinia pestis*. *F. tularensis*, *B. pseudomallei*, *L. innocua*, and *L. monocytogenes* were cultured with brain heart infusion broth containing 0.4% homocysteine. *V. cholerae* and *V. parahaemolyticus* were cultured with alkaline peptone water. The remaining bacteria were cultured with LB broth. *B. anthracis* Spore was prepared as previously described^18^. All the bacterial suspensions were collected at the logarithmic phase, rinsed in sterilized saline, serially diluted, and smeared on agar plates to measure the colony forming units (CFU). Subsequently, the bacterial concentrations were determined.

For the preparation of mAbs, 1 × 10^8^ CFU *F. tularensis* inactivated by formaldehyde was subcutaneously injected into Balb/c mice every two weeks for three routine immunizations, and one booster immunization with 5 × 10^8^ CFU inactivated *F. tularensis*. The spleen cells of Balb/c mice were collected, fused with myeloma cell (SP2/0), and cultured in a CO_2_ incubator. The supernatant of cell cultures was screened using ELISA coated with inactivated *F. tularensis*, and the positive cell cultures were cloned using the limited dilution method until 100% of the wells were positive. Subsequently, 1 × 10^8^ hybridoma cells were injected into Balb/c mice intraperitoneally to obtain the ascites, and mAbs against *F. tularensis* in ascites were screened by ELISA and purified using octanoic acid and saturated ammonium sulfate.

### Fabrication of the strip and detection

The mAbs (2 mg · mL^−1^) against *F. tularensis* and goat anti-mouse antibody were dispensed on nitrocellulose membrane as test line (T) and control line (C), respectively, at a speed of 1 μL · cm^−1^ and then dried at 37 °C. UCP-conjugating mAbs (1 mg · mL^−1^) were poured onto the glass fiber at a speed of 30 μL · cm^−1^ as conjugate release pad. The nitrocellulose membrane, conjugate release pad, and absorbent paper were adhered on a sticky base in sequence and then cut into 4 mm pieces to prepare into strips. The strips were placed into a plastic cartridge with sample-adding windows and result observation windows. For detection, the sample and sample-treating buffer were mixed at a ratio of 1:9, and100 μL of mixture was applied to each strip. After 15 min, the strips were scanned by a UPT biosensor.

### Sensitivity, linearity, precision, and specificity assessment

*F. tularensis* samples (10^3^–10^9^ CFU · mL^−1^) serially diluted with PB were analyzed by Ftu-UPT-LF in triplicate. The T/C ratios were measured for the negative and weak positive samples to evaluate the sensitivity of the assay. The stand quantification curve was plotted for *F. tularensis* ranging from 10^4^ CFU · mL^−1^ to 10^8^ CFU · mL^−1^, and the correlation coefficient of linear regression was obtained to assess the accuracy of quantification. The coefficient of variation for three trials was used to evaluate the precision of the strips.

The specificity of Ftu-UPT-LF was verified by 18 bacterial strains, which comprised four bacterial strains that were potential biowarfare/bioterrorism agents similar to *F. tularensis* (*B. anthracis* Spore, *B. melitensis* M55009, *B. pseudomallei*, and *Y. pestis*) and 14 bacterial strains that had a gastrointestinal infection route similar to *F. tularensis* (*E. coli* O157:H7, *L. innocua*, *L. monocytogenes*, *S. choleraesuis*, *S. dysenteriae*, *S. enteritidis*, *S. paratyphi* A, *S. paratyphi* B, *S. paratyphi* C, *S. typhi*, *S. typhimurium*, *V. cholerae* O1, *V. cholera* O139, and *V. parahaemolyticus*). With *F. tularensis* as control, 10^8^ and 10^9^ CFU · mL^−1^ of the 18 bacterial strains were detected by the strips.

### Evaluation of sample tolerance

To assess single-factor theoretical tolerance, HCl and NaOH, the mixture of KCl and NaCl, PEG20000 and glycerol, and BSA and casein were prepared into solutions with serial concentrations using deionized water. Subsequently, 10^3^–10^8^ CFU · mL^−1^ *F. tularensis* was spiked into these solutions and detected by Ftu-UPT-LF, and each experiment was repeated in triplicate.

To evaluate real sample tolerance, flour, fruit juice, gourmet powder, milk powder, putty powder, sucrose, and soil were dissolved in 0.03 mol · L^−1^ PB with final concentrations of 50, 100, and 200 mg · mL^−1^. Meanwhile, the viscera were ground by PB and prepared into 100, 200, and 400 mg · mL^−1^ homogenate. *F. tularensis* (10^3^ and 10^4^ CFU · mL^−1^) was spiked into the solution of these samples, and each sample was tested in triplicate.

### Tolerance evaluation of operation error

The liquid volumes were adjusted to simulate the operation error for evaluation with the standard operation as the control. To assess the influence of variations in the sample and sample-treating buffer volumes, the ratios of sample and sample-treating buffer were set as 5:90, 20:90, and 30:90 and 10:70, 10:110, and 10:130, respectively. Subsequently, 100 μL mixtures were loaded onto the strips. For tolerance evaluation of loading mixture error, samples and sample-treating buffer were mixed at a routine ratio of 10:90, and 70, 110, and 130 μL were applied to the strips.

## Additional Information

**How to cite this article**: Hua, F. *et al*. Development and evaluation of an up-converting phosphor technology-based lateral flow assay for rapid detection of *Francisella tularensis*. *Sci. Rep*. **5**, 17178; doi: 10.1038/srep17178 (2015).

## Figures and Tables

**Table 1:** Tolerance limits of Ftu-UPT-LF assay to pH, ion strength, viscosity, biomacromolecule, and real samples.

Interference Factor		Ftu-UPT–LF
pH value	HCl	≤0.01 mol · L^−1^ (pH 2)
	NaOH	≥0.1 mol · L^−1^ (pH 13)
Ion strength	NaCl + KCl	≥2 mol · L^−1^
Viscosity	PEG20000	≤50 mg · mL^−1^
	Glycerol	≥20% (v/v)
Biomacromolecule	BSA	≥400 mg · mL^−1^
	Casein	≥80 mg · mL^−1^
Powder	Flour	≥200 mg · mL^−1^
	Fruit juice	≥200 mg · mL^−1^
	Gourmet powder	≥200 mg · mL^−1^
	Milk powder	≤50 mg · mL^−1^
	Putty powder	≥200 mg · mL^−1^
	Soil	≤100 mg · mL^−1^
	Sucrose	≥200 mg · mL^−1^
Viscera	Fresh heart	≥400 mg · mL^−1^
	Fresh liver	≥400 mg · mL^−1^
	Fresh lung	≥400 mg · mL^−1^
	Fresh spleen	≥400 mg · mL^−1^
	Decomposed heart	≥400 mg · mL^−1^
	Decomposed liver	≥400 mg · mL^−1^
	Decomposed lung	≥400 mg · mL^−1^
	Decomposed spleen	≥400 mg · mL^−1^
